# Supplementary material for: Complexome profiling of the Chlamydomonas psb28 mutant reveals TEF5 as an early PSII assembly factor
Source: Plant Cell. 2025 Mar 17;37(6):koaf055. doi: 10.1093/plcell/koaf055 (PMC12164590; doi:10.1093/plcell/koaf055)
Supplement: koaf055_Supplementary_Data [file koaf055_supplementary_data.zip › psb28_interactive.zip!/plots/intensity/qualityPlots/peptides/_Cre09.g405450.t1.2.html]

### Cre09.g405450.t1.2

Trivial name:   
SeqCoverage [median]: 27.90  
Mass: 14.25 kDa  
  
 

| Sequence | MissedCleavage | Mass [Da] | wtIntensity | muIntensity | Score |
| --- | --- | --- | --- | --- | --- |
| EFHLFDTVLSK | 0 | 1334.69 | 2144.26 | 0.00 | 80.75 |
| TYNGKPVPPELQGADYITYAGVTMK | 1 | 2712.35 | 0.00 | 1199.40 | 45.10 |
|  |  |  | 2144.3 | 1199.4 |  |
